# Deep Convolutional Neural Network–Based Computer-Aided Detection System for COVID-19 Using Multiple Lung Scans: Design and Implementation Study

**DOI:** 10.2196/27468

**Published:** 2021-04-26

**Authors:** Mustafa Ghaderzadeh, Farkhondeh Asadi, Ramezan Jafari, Davood Bashash, Hassan Abolghasemi, Mehrad Aria

**Affiliations:** 1 Department of Health Information Technology and Management School of Allied Medical Sciences Shahid Beheshti University of Medical Sciences Tehran Iran; 2 Department of Radiology Baqiyatallah University of Medical Sciences Tehran Iran; 3 Department of Hematology and Blood Banking School of Allied Medical Sciences Shahid Beheshti University of Medical Sciences Tehran Iran; 4 Pediatric Congenital Hematologic Disorders Research Center Shahid Beheshti University of Medical Sciences Tehran Iran; 5 Department of Computer Engineering, Faculty of Electrical and Computer Engineering Shiraz University Shiraz Iran

**Keywords:** artificial intelligence, classification, computer-aided detection, computed tomography scan, convolutional neural network, coronavirus, COVID-19, deep learning, machine learning, machine vision, model, pandemic

## Abstract

**Background:**

Owing to the COVID-19 pandemic and the imminent collapse of health care systems following the exhaustion of financial, hospital, and medicinal resources, the World Health Organization changed the alert level of the COVID-19 pandemic from high to very high. Meanwhile, more cost-effective and precise COVID-19 detection methods are being preferred worldwide.

**Objective:**

Machine vision–based COVID-19 detection methods, especially deep learning as a diagnostic method in the early stages of the pandemic, have been assigned great importance during the pandemic. This study aimed to design a highly efficient computer-aided detection (CAD) system for COVID-19 by using a neural search architecture network (NASNet)–based algorithm.

**Methods:**

NASNet, a state-of-the-art pretrained convolutional neural network for image feature extraction, was adopted to identify patients with COVID-19 in their early stages of the disease. A local data set, comprising 10,153 computed tomography scans of 190 patients with and 59 without COVID-19 was used.

**Results:**

After fitting on the training data set, hyperparameter tuning, and topological alterations of the classifier block, the proposed NASNet-based model was evaluated on the test data set and yielded remarkable results. The proposed model's performance achieved a detection sensitivity, specificity, and accuracy of 0.999, 0.986, and 0.996, respectively.

**Conclusions:**

The proposed model achieved acceptable results in the categorization of 2 data classes. Therefore, a CAD system was designed on the basis of this model for COVID-19 detection using multiple lung computed tomography scans. The system differentiated all COVID-19 cases from non–COVID-19 ones without any error in the application phase. Overall, the proposed deep learning–based CAD system can greatly help radiologists detect COVID-19 in its early stages. During the COVID-19 pandemic, the use of a CAD system as a screening tool would accelerate disease detection and prevent the loss of health care resources.

## Introduction

In 2020, the rapid global spread of COVID-19 made the World Health Organization declare the first pandemic of the 21st century, with the highest level of alert worldwide. Based on the WorldMeters statistics, until January 5, 2021, more than 86 million people worldwide contracted this disease, with more than 1,870,000 confirmed deaths due to COVID-19. Early detection of COVID-19 is essential not only for patient care but also for public health by ensuring patients’ isolation and controlling disease spread [1,2]. The first and most important step to control this pandemic is the rapid detection of infected patients and monitoring of positive cases. Various diagnostic methods for the rapid detection of COVID-19 have been introduced by different studies and by the WHO, with the reverse transcription–polymerase chain reaction (RT–PCR) test being the most prominent diagnostic method. Although RT–PCR is the gold standard for COVID-19 detection, owing to its time-intensiveness and high cost, infected individuals, as a source of transmission, can transmit the virus to many people while they are waiting to receive the results of their RT–PCR test. Moreover, previous studies have reported that the RT–PCR test has a high false-negative rate; this is a major limitation of this diagnostic test and reduces its sensitivity. Furthermore, this leads to delayed detection, treatment, and—in advanced stages of the disease—an increased mortality rate [3-8]. A high influx of patients at diagnostic centers during the pandemic has led to excessive use of resources and a shortage of RT–PCR test kits. Regardless of the need of RT–PCR tests for suspected individuals, the multiple repeats of these tests for patients have imposed a heavy burden on health care sources. The time-consuming nature of laboratory tests, coupled with the molecular and nonspecific nature of serological tests, has necessitated the use of a cheaper test focusing on findings in the lung tissue. As major lung health monitoring tools, radiological tests have attracted the attention of clinical specialists. For COVID-19 evaluation, computed tomography (CT) is a more sensitive and specific detection method than chest X-ray imaging, and, in many cases, lung involvement and ground-glass opacities (GGO) can be viewed on CT even before the onset of clinical symptoms and before obtaining positive results on an RT–PCR test. This implies that, in many cases, before the emergence of the first clinical symptoms and a positive RT–PCR finding, the complications of COVID-19 can be detected in the lungs. Based on previous reports and the WHO’s recommendations, chest CT has emerged as a valuable tool for the early detection and triaging of individuals suspected with COVID-19 [4,9,10]. In a study on 1014 patients with COVID-19, CT enabled more sensitive detection than RT–PCR [11]. Despite the success of this radiological modality in detecting COVID-19–related lung damage, certain problems are associated with its use. Despite the WHO’s recommendations, chest CT findings are normal in some patients at the outset of the disease, and this lends a negative predictive value to CT alone. The low-specificity of CT can deter disease detection in non–COVID-19 cases. In addition, ionizing radiation from the CT scanner can cause problems to patients who require multiple CT scans during the course of their disease [12-16]. In the past decade, numerous computer-based methods have been employed for improving the efficiency of medical imaging techniques. One such method is the use of machine learning algorithms, which has had remarkable success in medical imaging. Among different types of machine learning methods, deep learning models have achieved high precision in machine vision tasks rapidly after the emergence of COVID-19. Convolutional neural networks (CNNs) have high potential for feature extraction and analysis. Upon the emergence of COVID-19, and owing to the limitations of diagnostic tests, numerous machine learning techniques have been adopted to improve the precision of diagnostic methods. Table 1 lists some relevant studies.

**Table 1:** Studies evaluating machine learning algorithms used for COVID-19 detection.

Study (country)	Study objective	Population	Models used	Evaluation results
Ni et al (China) [15]	Automatic detection	14,531	Convolutional multiview feature pyramid network with positron-aware attention and a 3D U-Net	F_1_ score=97% Sensitivity=100%
Wang et al (China) [17]	Diagnostic and prognostic analysis	5372	Densenet121-feature pyramid network	Area under the receiver operating characteristic curve=87%-88% Sensitivity=80.3%-79.35%
Hasan et al (Iraq) [18]	Diagnosis (classification)	321	Long short-term memory classifier	Accuracy=99.68%
Pathak et al (India) [19]	Classification (detection)	852	ResNet-50	Accuracy=93.01%
Ardakani et al (Iran) [20]	Detection	194	10 pretrained convolutional neural networks: AlexNet, VGG-16, VGG-19, SqueezeNet, GoogleNet, MobileNet-V2, ResNet-18, ResNet-50, ResNet-101, and Xception	Best performance: ResNet-101 and Xception Sensitivity (ResNet-101)=100% Sensitivity (Xception)=98.04% Specificity (ResNet-101): 99.02% Specificity (Xception)=100% Accuracy (ResNet-101)=99.51% Accuracy (Xception)=99.02%
Li et al (China) [21]	Automatic detection	4356	RestNet50 as the backbone of the main model	Sensitivity=90% Specificity=96%
Mei et al (United States) [22]	Rapid diagnosis	905	Inception-ResNet-V2	Correctly identified 17 of 25 (68%) patients with COVID-19
Song et al (China) [23]	Diagnosis	227	Bidirectional generative adversarial network	Sensitivity=85% Specificity=88%

## Methods

### Study Overview

Based on the success of CNNs in machine vision tasks, we designed and implemented a model for the classification of CT images of individuals with and those without COVID-19 through a deep neural network based on a Neural Search Architecture Network (NASNet) [24] feature extractor.

### Data Set

**Figure 1:**
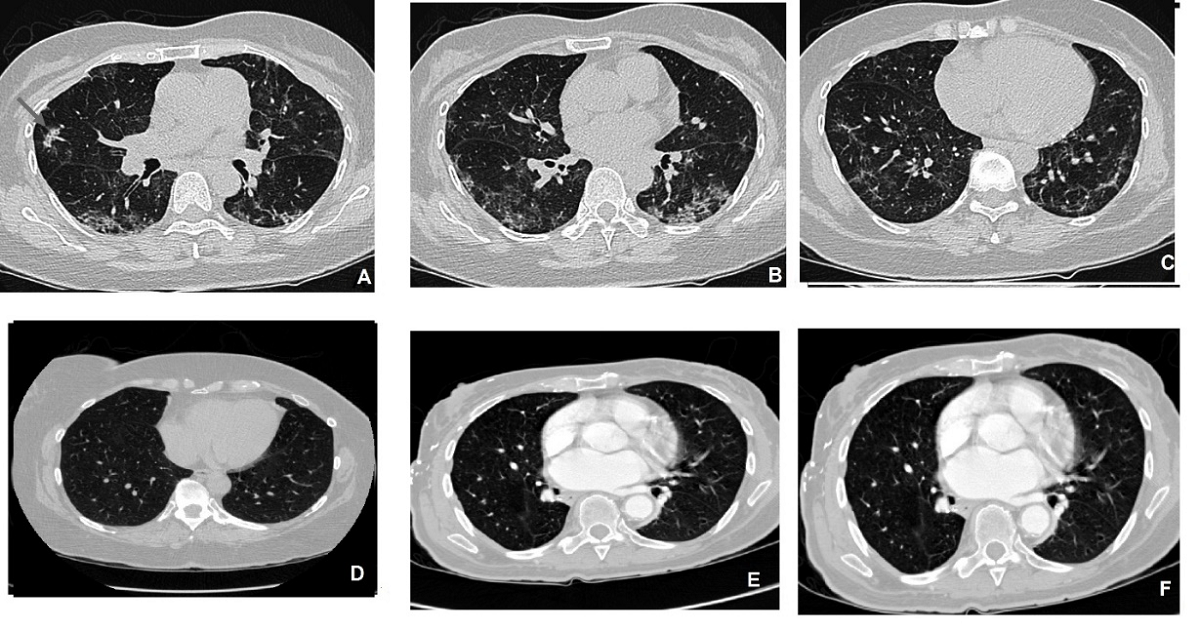
Axial computed tomography scan slices of the lung. (A, B, C) Non–COVID-19 cases including those of pneumonia and healthy individuals; (D, E, F) infected lungs of patients with COVID-19.

The data set comprised 10,153 CT scans, of which 7644 belong to 190 patients with COVID-19 and 2509 belong to 59 people without COVID-19, including those with pneumonia and otherwise healthy individuals who visited the hospital owing to a suspicion of COVID-19 [25]. All these images were collected from the radiology centers of teaching hospitals in Tehran, Iran. The disease status in suspected individuals was confirmed in this set after an RT–PCR test. Figure 1 shows the CT scans of some patients with COVID-19 and their counterparts with suspected disease.

### Proposed Method

To detect COVID-19 in patients at an early stage of the disease from multiple lung CT scans, a state-of-the-art model based on a NASNet CNN feature extractor was proposed. Based on the proposed model, a computer-aided detection (CAD) system was designed.

### Data Preparation and Preprocessing

For data preparation, lung CT scans were first received in the Digital Imaging and Communications in Medicine format as the output of the picture archiving and communications system of a diagnostic center. In the preprocessing stage, the images were converted to the commonly used JPG format, and the order of the color channels was changed from the default BGR to RGB to prepare the images for processing.

Based on the literature, the success of medical image visual tasks in deep learning is not merely attributed to CNN models; rather, a major part of this success results from image preprocessing [26]. Data normalization to maintain the integrity of the images was performed as the first step of preprocessing, which plays a key role in the analysis of CT scans [27]. To this end, first, the pixel-level global mean (SD) values were calculated for all the images; thereafter, the data were normalized using the following equation:

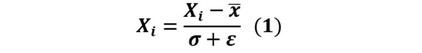



where 
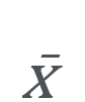
 is the global mean of the image set *X*, *σ* is the SD, and *ε*=1*e*–10 is an insignificant value to prevent the denominator from becoming 0.

After normalization, for standardizing the images to achieve a unified scale for the input of the deep neural network, the values of the pixels of each image were scaled by transferring to (0,255) and then by transforming to the (0,1) interval, such that the images would be standard during training. Since CNNs depend on a large number of data to enhance their efficiency and prevent model over-fitting [28,29]; at this stage, data were augmented for the training data set through random rotation, contrast alteration, illumination alteration, and gamma correction.

All the images of the sets were shuffled such that the network would not necessarily see the data of only a certain class during training, and each batch would include images with different labels belonging to both COVID-19 and non–COVID-19 classes. The dimensions of the input images were changed to 224×224×3; however, this method can be used on images with any other dimensions. The data set with the 64:16:20 ratio was randomly divided among the training, validation, and test sets, respectively, whereby 20% of the data were allocated to the test set, the remaining 80% to the training set, and 20% of the training set was assigned to the validation set.

### Feature Extraction and Classification

**Figure 2:**
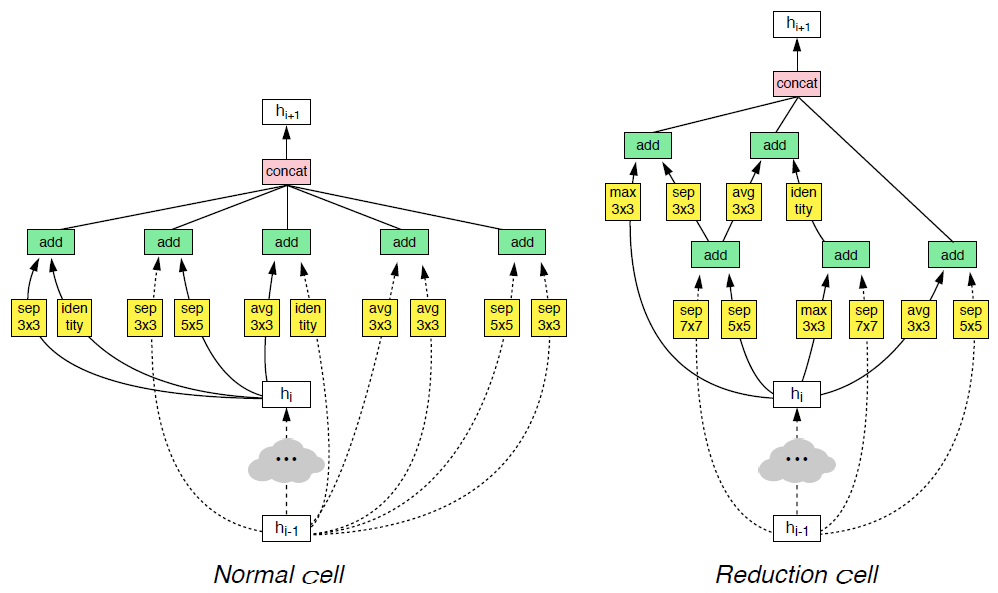
Architecture of the NASNet’s convolutional cells with B=5 blocks. The input (white) is the hidden state from previous activations (or input image). The output (pink) is the result of a concatenation operation across all resulting branches. Each convolutional cell is the result of B blocks. A single block corresponds to 2 primitive operations (yellow) and a combination operation (green) [23].

Convolutional layers were used in the feature extraction block. Immediately after each Conv2D layer, the useful statistics were collected using the Max-Pooling module and, after normalizing them using batch normalization, passed to the next CNN block. To prevent model overfitting, in addition to batch normalization, weight regularization and dropout methods were also applied. For regularization, the Euclidean norm (L2) was used with different coefficient values in the (0.001-0.01) interval after activation using the LeakyReLU activation function and dropout of 20%-30% of the weights. Inspired by the transfer learning approach, for better feature extraction, the preliminary blocks of the pretrained NASNetLarge network were used. NASNet has a scalable architecture for image classification and consists of 2 repeated motifs termed the normal cell and the reduction cell. Figure 2 illustrates the architecture of these convolutional cells. All parameters were initialized using the weights obtained from fitting NASNetLarge on the ImageNet data set. After the feature extraction block, the weights were transferred to 3 dense or fully connected layers by using a global average pooling flatten layer in the form of a 1-dimensional tensor.

In these layers, batch normalization, regularization, and weight dropout were performed as well. The first dense layer used a ReLU activation function, the next layer utilized a LeakyReLU, and the last layer, which actually is the classifier layer, employed a Softmax multiclass activation function.

During the training process, in the first phase, the feature extraction block was frozen and contained nontrainable parameters, and Adam was used as the optimizer. In this phase, the initial learning rate of 1*e*–3 and the binary cross-entropy loss function were used. If validation loss remained stable in every 10 epochs, the learning rate would be reduced by 20% to a minimum of 1*e*–6 in case of no further improvement. If the validation loss remained stable up to 20 epochs, the process of training would stop. Eventually, only the best weights were saved. After training the dense layers, in the second phase the feature extraction block was unfreezed, and the network—this time fully trainable—was fitted once more on the same data; in this phase, the stochastic gradient descent optimizer with the initial learning rate of 1*e*–4 was utilized. The batch size was 32; the number of epochs in the first phase was assumed to be 200, and 1000 iterations were considered in the second phase.

### CAD System Based on the Proposed Model

**Figure 3:**
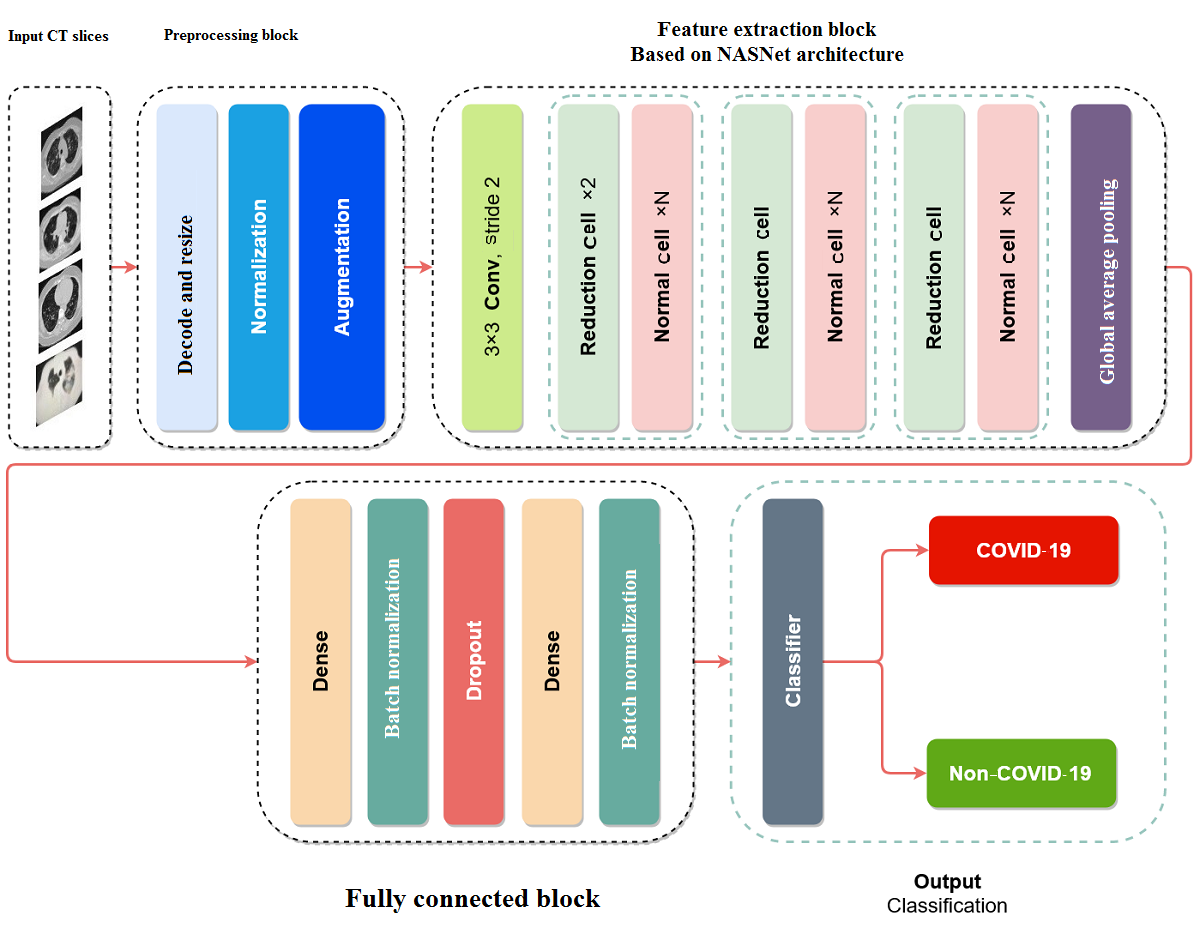
Proposed deep convolutional neural network–based CAD system for COVID-19 detection using multiple lung computed tomography scans. CT: computed tomography.

Many studies have recommended the use of CT scans for COVID-19 detection, many of which have used machine learning–based computer methods to enhance the results of chest CT. All machine learning methods have attempted to detect COVID-19 in the images with a single CT slice [10,12,30-33]; however, in real time, radiologists confirm or reject COVID-19 on the basis of overall slices of a patient’s CT scan. This study aimed to design a computer-aided diagnostic system to detect COVID-19 with multiple CT images for each person. In the CAD system designed on the basis of the proposed model, 4 CT slices were obtained from a person suspected with COVID-19 and the system estimates the final result from the output average mean of classifying all the slices. However, the proposed system can receive a different number of slides and does not depend on the number of inputs. This increases the reliability of the results obtained from the proposed model. Figure 3 provides a schematic representation of the proposed model.

**Figure 4:**
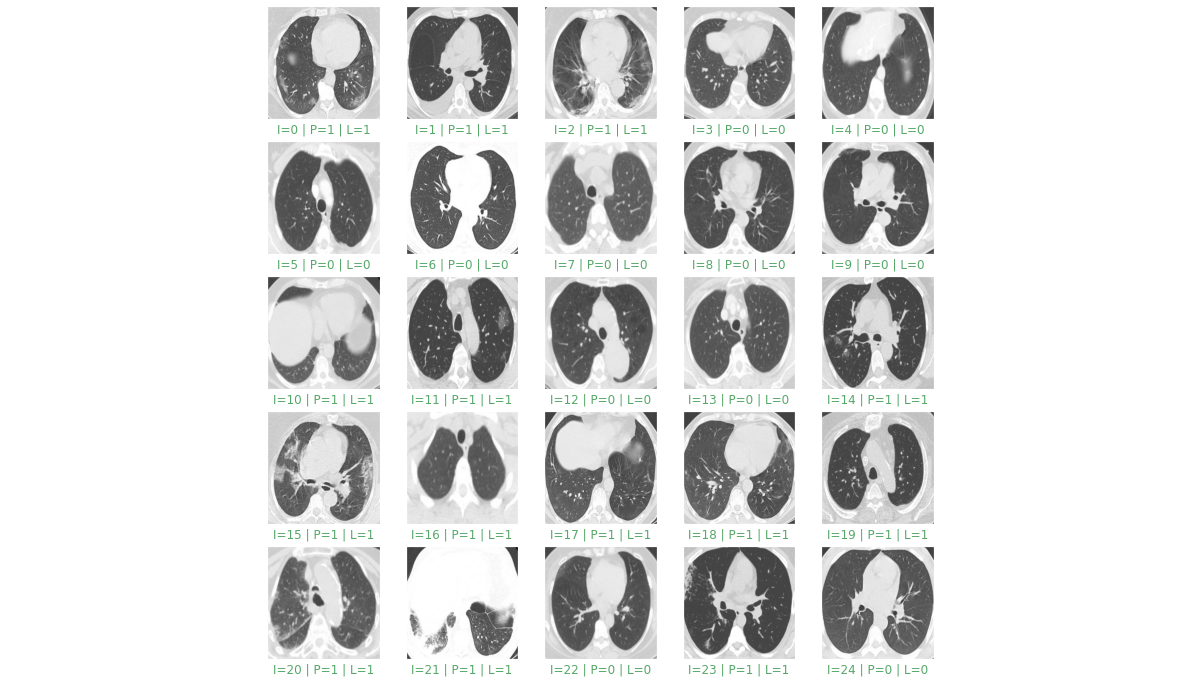
Results of the detection on the first 25 samples from the test set. “I” is the image index, “P” is the predicted value, and “L” is the grand truth label. Green indicates correct detection and red indicates incorrect detection.

**Figure 5:**
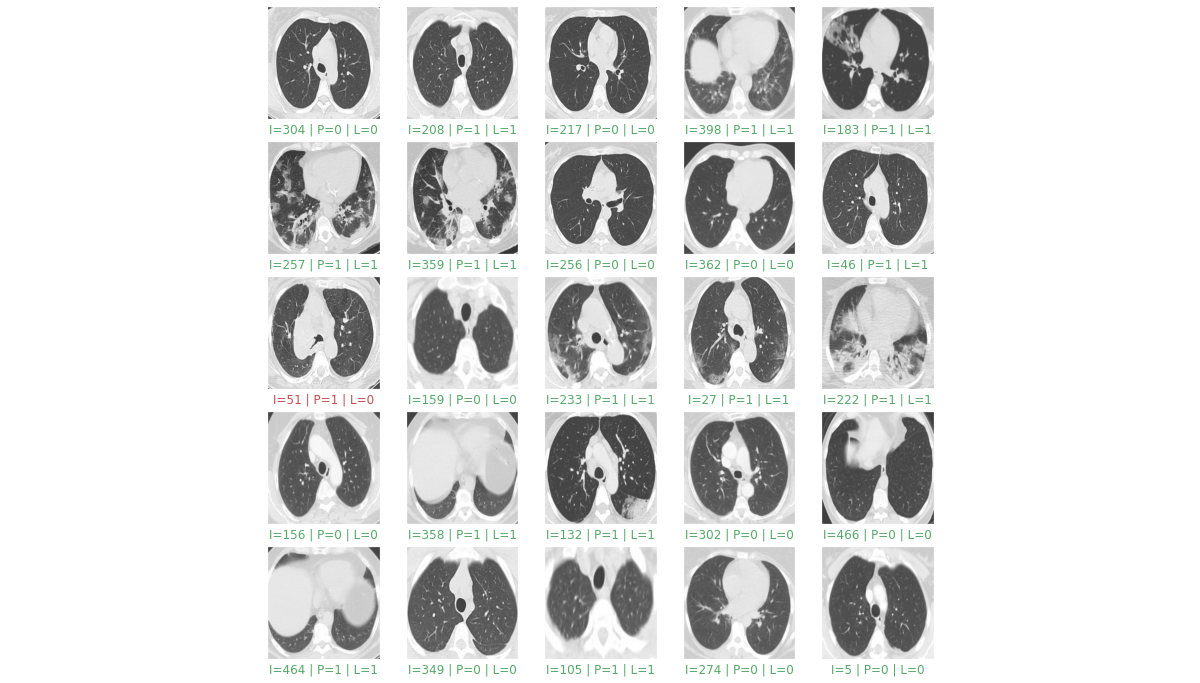
Results of detection on 25 random samples from the test set. “I” is the image index, “P” is the predicted value, and “L” is the grand truth label. Green indicates correct detection and red indicates incorrect detection.

In the experiments, the proposed model successfully detected all the cases of COVID-19 with high accuracy and differentiated all the positive and negative cases without any discernible error. Figures 4 and 5 display the performance of the proposed model by presenting the results of detection on the first 25 samples and 25 random ones from the test set, respectively.

In the case of COVID-19, the lack of an early and accurate diagnosis leads to the spread of the disease among other individuals, which has irreversible effects on the control of the pandemic. The proposed CAD system, which detects cases of COVID-19 from multiple CT slices can perform more accurately because, owing to the error of not showing the area of the GGO in some CT slices, the data of many slices may have been missed. Moreover, in models that detect COVID-19 from a single slice, there is a risk of error in viewing the infected area of the lung or ROI owing to operator errors, the angle of the slices, or problems with the CT scanner tube. Thus, by using a multislice CAD system, the disease can be detected in its early stages, and the initial signs of lung involvement can be discovered with maximum precision.

### Implementation

The proposed method was implemented using the Python programming language by Keras, which is a high-level library for TensorFlow machine learning framework which also utilized a Compute Unified Device Architecture deep learning network library for parallel processing on the graphics processing unit. The computer system had an Intel Core i7 7700K CPU, 32 GB RAM, and an Nvidia T4 GPU accelerator. Implementation codes and the pretrained model are available on GitHub [34].

## Results

### Metrics

To quantitatively evaluate the performance of the proposed method, the sensitivity, specificity, accuracy, and *F*_1_ score evaluation criteria were determined on the basis of the model’s performance by using a confusion matrix. Here, sensitivity was defined as the ratio of COVID-19 cases correctly detected by the model to all the actual COVID-19 cases. Specificity was defined as the ratio of the non–COVID-19 cases correctly detected by the model to all the actual non–COVID-19 cases. Moreover, accuracy was defined as the rate of all the COVID-19 and non–COVID-19 cases accurately detected on the basis of the CT images.

### Experimental Results and Evaluation

In this study, we used traditional measures to evaluate the performance of the proposed model, using a confusion matrix. Based on this confusion matrix, the specificity and sensitivity in measuring and analyzing the performance of the proposed CAD model were calculated, where specificity was defined as the ability of the classifier to correctly identify individuals without COVID-19 (true-negative rate). Sensitivity was defined as the classifier’s ability to identify individuals with COVID-19 correctly (true-positive rate). These evaluations were performed using the following equations:

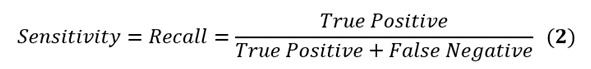

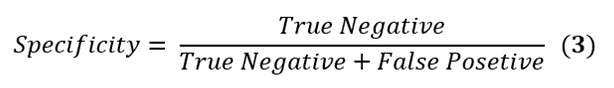

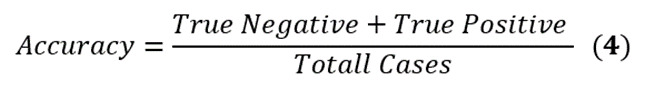







**Figure 6:**
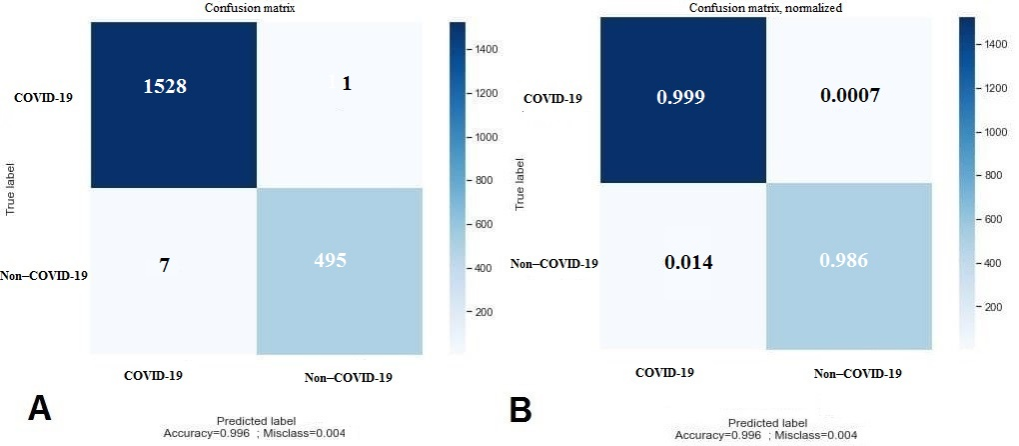
(A) Confusion matrix and (B) normalized confusion matrix of the model performance for the test data.

Figure 6 shows the confusion matrix for the evaluation in the test set for 2 classes. The evaluation criteria based on the confusion matrix are provided in Table 2.

**Table 2:** Performance of the proposed method in the test data set.

Metric	Value
Sensitivity	99.9
Specificity	98.6
Accuracy	99.6

**Figure 7:**
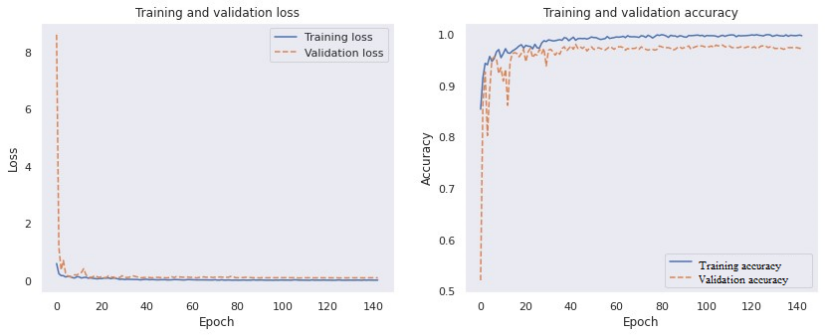
Training and validation loss and accuracy.

The learning curve of the proposed model for the training and validation sets is illustrated in Figure 7. On assessing the behavior of the proposed model in handling new validation data, we observed that with increased epochs, the model had a lower error rate, and thus enhanced accuracy for the unknown data, which suggests that the model has high potential for detecting new cases of COVID-19 from the CT scans. The mean square error in detecting all the COVID-19 and non–COVID-19 cases from among the test set images was 0.003938, which was significantly lower than that reported in previous studies.

The metrics of positive predictive value and negative predictive value, as well as the *F*_1_ score, for the proposed model are shown in Table 3.

**Table 3:** Evaluation of the proposed model for the test set.

Metric	Value (%)
Positive predictive value = True positive / Positive calls	99.8%
Negative predictive value = True negative / Negative calls	99.7%
F_1_ score = (True positive / True positive) + 0.5 (False positive + False negative)	99.8%

To evaluate the performance of the proposed model further in real-life applications and present a comparable evaluation, we tested our model on a publicly available and well-known data set [30] using the cross–data set evaluation approach. The results shown in Table 4 compare the proposed method with other state-of-the-art approaches, including traditional deep neural networks and pretrained networks [30-33,35].

**Table 4:** Comparison of the performance of different models for detecting COVID-19 using various evaluation metrics.

Model	Evaluation Metrics			
	Accuracy	Precision	Recall	*F*_1_ score
SqueezeNet	95.1	94.2	96.2	95.2
ShuffleNet	97.5	96.1	99.0	97.5
GoogleNet	91.7	90.2	93.5	91.8
VGG-16	94.9	94.0	95.4	94.9
AlexNet	93.7	94.9	92.2	93.6
ResNet50	94.9	93.0	97.1	95.0
Xception	98.8	99.0	98.6	98.8
AdaBoost	95.1	93.6	96.7	95.1
Decision Tree	79.4	76.8	83.1	79.8
Explainable deep learning [30]	97.3	99.1	95.5	97.3
DenseNet201 [31]	96.2	96.2	96.2	96.2
Modied VGG19 [32]	95.0	95.3	94.0	94.3
COVID CT-Net [33]	90.7	88.5	85.0	90.0
Contrastive Learning [35]	90.8	95.7	85.8	90.8
Proposed	99.4	99.6	99.8	99.5

## Discussion

### Principal Findings

RT–PCR is the definitive method for diagnosing COVID-19. However, the nucleic acid test is very time-consuming, and sputum analysis may take several days. This test’s high cost and low sensitivity have caused major problems to health care systems during the pandemic. Consequently, people with false-negative findings on RT–PCR have been a source of virus transmission and have spread the virus to others. When the WHO emphasized the need to increase diagnostic tests and comprehensively evaluate suspected individuals, physicians and health care systems were encouraged to utilize cheaper and faster tests [36-38]. When attempting to detect COVID-19 in its initial stages, a lung CT scan does not always demonstrate the lung consolidation areas, and no GGO findings are observed in many cases. Machine learning models can enhance the efficiency of radiological diagnostic methods and serve as a suitable alternative to the RT–PCR test. The core of the CAD system designed in this study is based on a deep CNN architecture and uses an input with 4 slices. NASNet was utilized here because it could determine the best architecture for feature engineering [24]. No previous study has employed this technological model for analyzing the CT scans of individuals suspected with COVID-19. Further examination of medical image processing revealed the remarkable performance of this model in image feature extraction. This study achieved maximum sensitivity and precision in detecting COVID-19 compared to previous studies. Considering the algorithm and the use of multiple chest CT scan slices for a single patient, the proposed system can be employed at diagnostic centers as a reliable method to detect individuals with COVID-19 with high precision in the early stages of the disease. In the future, this CAD can be included in the picture archiving and communications systems of radiology wards to achieve an automated and more efficient diagnosis.

### Conclusions

Using the CAD system for detecting COVID-19 during the pandemic minimizes the time of image interpretation and consequently the number of patients waiting at radiology centers. Furthermore, by increasing the number of images produced by the CT scanner and increasing the population size, better classification results for differentiating positive and negative cases can be expected.

## Abbreviations

CNN: convolutional neural network
CAD: computer-aided detection
CT: computed tomography
NASNet: neural search architecture network
GGO: ground-glass opacities
RT–PCR: reverse transcription–polymerase chain reaction
WHO: World Health Organization
